# Comparison of Medical Input on Older Adult Versus General Adult Psychiatric Wards – A Retrospective Study

**DOI:** 10.1192/bjo.2025.10501

**Published:** 2025-06-20

**Authors:** Roberta Manuel, Stephanie Wan, Ivan Shanley

**Affiliations:** 1 Mid and South Essex NHS Foundation Trust, Essex, United Kingdom; 2 Partnership University NHS Foundation Trust, Essex, United Kingdom

## Abstract

**Aims:** This study examines the level of medical input for physical conditions provided to older age psychiatry patients compared with general adult ward patients. The assessment focuses on the frequency of medical reviews, the reasons for these reviews (e.g. falls, infections, heart failure), and National Early Warning Score (NEWS) escalations. The aim was to identify disparities in medical involvement and determine whether increased staffing is necessary in older age psychiatry wards.

**Methods:** The study included patients from an old age psychiatry ward (aged 65 and above) and a general adult ward (aged 18–64) over a one-month period. Data were collected from medical continuation sheets, patient records, and NEWS score documentation. Key variables included the number of medical reviews, reasons for these reviews, and the frequency of NEWS score escalations (≥5). A total of 46 patients were included in this study. Comparative statistical analysis was conducted to quantify medical input disparities between the two wards.

**Results:** The analysis revealed significant differences in medical input between the two wards. The mean age of patients in the old age psychiatry ward was 73.2 years, compared with 35.1 years in the general adult ward. Older patients required substantially more medical reviews, with a mean of 5.05 per patient per month, whereas younger adults had a mean of 0.91 review per patient (a significant difference, P<0.0001). The most common indications for medical reviews in older adults included falls, infections, heart failure, and respiratory distress. In contrast, younger adults primarily presented with milder complaints such as gastrointestinal issues and minor injuries. NEWS score escalations (≥5) occurred in 9% of older patients, compared with none in the younger cohort. Additionally, 21.7% of older patients required emergency department visits, significantly higher than the 4.3% observed in the general adult ward.

**Conclusion:** This study confirms a significantly higher requirement for medical input for older age psychiatry patients compared with general adult ward patients. It is recommended that additional medical staffing provision is considered in old age psychiatry wards. Additionally, regular training on NEWS escalation management and interdisciplinary collaboration between psychiatry and medical teams may improve patient outcomes.

